# Supplementary material for: αO-Conotoxin GeXIVA Inhibits the Growth of Breast Cancer Cells via Interaction with α9 Nicotine Acetylcholine Receptors
Source: Mar Drugs. 2020 Apr 7;18(4):195. doi: 10.3390/md18040195 (PMC7231225; doi:10.3390/md18040195)
Supplement: Supplementary file 1 [file marinedrugs-18-00195-s001.pdf]

Supplementary Materials:

αO-Conotoxin GeXIVA Inhibits the Growth of Breast Cancer Cells via Interaction with α9 Nicotine Acetylcholine Receptors

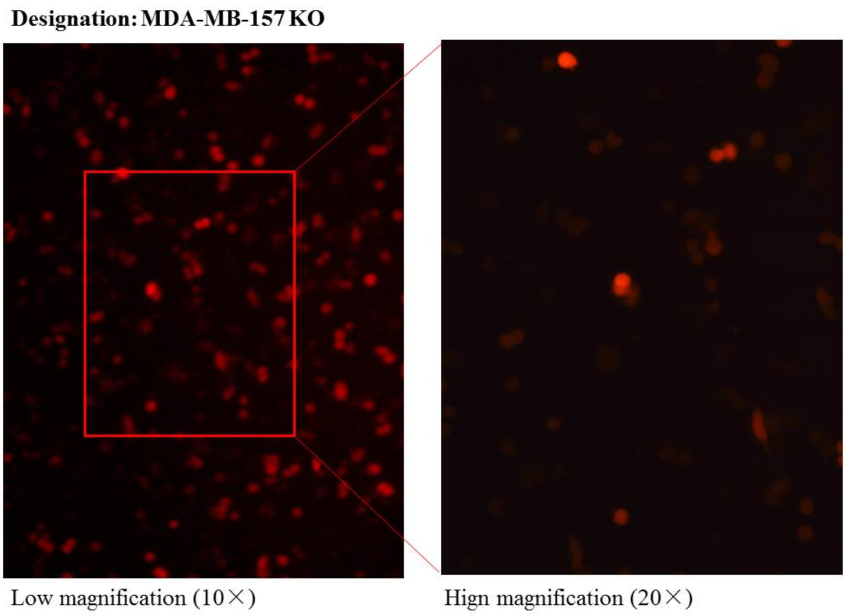

Figure S1. The α9-nAChR CRISPR/Cas9 KO plasmid ad HDR plasmid co-transfected into MDA-MB-157 cell line confirmed by detection of the red fluorescent protein (RFP) via fluorescent microscopy.

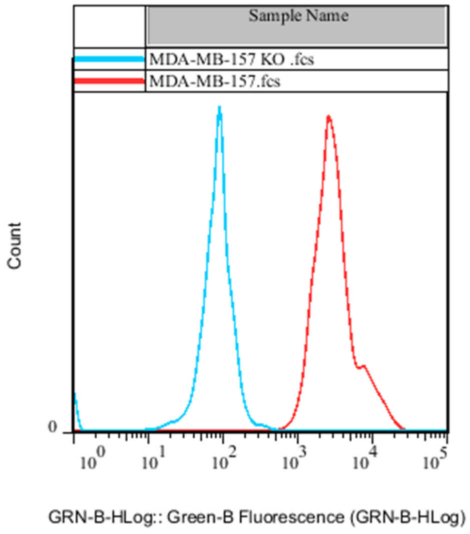

Figure S2. Representative histograms of cell distribution according to intensity of FITC-labeled antibody against the α9-nAChR for untreated cells (left) and cells with the knocked out α9-nAChR expression by CRISPR/Cas9 (right).
